# Considerations for Novel COVID-19 Mucosal Vaccine Development

**DOI:** 10.3390/vaccines10081173

**Published:** 2022-07-23

**Authors:** Wael Alturaiki

**Affiliations:** Department of Medical Laboratory Sciences, College of Applied Medical Sciences, Majmaah University, Majmaah 11952, Saudi Arabia; w.alturaiki@mu.edu.sa; Tel.: +966-555122264

**Keywords:** iBALT, BAFF, APRIL, CXCL13, CCL19, CCL21, COVID-19, B cell, T cell, COVID-19, mucosal vaccine

## Abstract

Mucosal surfaces are the first contact sites of the severe acute respiratory syndrome coronavirus 2 (SARS-CoV-2). Most SARS-CoV-2 vaccines induce specific IgG responses but provide limited mucosal immunity. Cytokine B-cell activation factor (BAFF) and A proliferation-inducing ligand (APRIL) in the tumor necrosis factor (TNF) superfamily play key immunological functions during B cell development and antibody production. Furthermore, homeostatic chemokines, such as C-X-C motif chemokine ligand 13 (CXCL13), chemokine (C–C motif) ligand 19 (CCL19), and CCL21, can induce B- and T-cell responses to infection and promote the formation of inducible bronchus-associated lymphoid tissues (iBALT), where specific local immune responses and memory cells are generated. We reviewed the role of BAFF, APRIL, CXCL13, CCL19, and CCL21 in the activation of local B-cell responses and antibody production, and the formation of iBALT in the lung following viral respiratory infections. We speculate that mucosal vaccines may offer more efficient protection against SARS-CoV-2 infection than systematic vaccines and hypothesize that a novel SARS-CoV-2 mRNA mucosal vaccine using BAFF/APRIL or CXCL13 as immunostimulants combined with the spike protein-encoding mRNA may enhance the efficiency of the local immune response and prevent the early stages of SARS-CoV-2 replication and the rapid viral clearance from the airways.

## 1. Introduction

In late 2019, a novel virus that caused a wide range of respiratory symptoms, ranging from mild to critical cases, known as severe acute respiratory syndrome coronavirus 2 (SARS-CoV-2), emerged and caused a global pandemic [1]. According to the WHO Health Organization’s coronavirus disease 2019 (COVID-19) dashboard, over 500 million confirmed cases of COVID-19 have been reported [2]. Furthermore, in response to this event, several academic institutions, research centers, and biotech companies worldwide are in a race against time to develop, evaluate, and approve several vaccines against SARS-CoV-2 infection [3]. Furthermore, most currently licensed vaccines are administered via injection and designed to induce specific IgG responses that can neutralize the virus and prevent viremia and COVID-19 symptoms. However, their abilities to elicit mucosal immunity are limited [4]. Thus, developing an effective vaccine that is delivered directly via the mucosal route may enhance and activate the local mucosal immune response against SARS-CoV-2 infection, which is important for preventing early viral replication and airway shedding. The respiratory tract is initially in direct contact with the outside environment; therefore, there is a risk of exposure to pathogens that can cause lung infections and even develop into life-threatening diseases, particularly when they lead to extreme recruitment and the activation of immune cells that can cause increased inflammation of the airways [5].

Furthermore, the SARS-CoV-2 infection is initiated after the interaction of the spike protein of the virus with the angiotensin-converting enzyme two receptors (ACE-2), which are expressed in the upper and lower respiratory tract, specifically in the bronchial epithelial cells. These cells, in turn, respond by producing important inflammatory cytokines and chemokines, facilitating the recruitment of multiple immune cell types to the site of infection, which is critical for viral clearance and the establishment of antiviral immune memory cells. The failure of virus removal will lead to uncontrolled excessive inflammation of the airways and cause lung injury [6,7,8]. Although the COVID-19 pandemic has been ongoing for more than two years, mucosal immunity against SARS-CoV-2 has received little attention. Moreover, mucosal immunity plays an important role in defense against the early stages of SARS-CoV-2 infection by producing a local mucosal secretory IgA response that can impede viral replication and shedding in the airways [4,6]. Consequently, the demand for mucosal vaccines has been emphasized recently [9], and numerous studies have addressed this option during the current pandemic [10,11,12,13]. In addition, a recent study reported that intranasal vaccines are safer and offer potent complementary protection as a booster compared to conventional systemic vaccines [6]. Resting lung immune cells play an essential role in protecting against invading pathogens by removing inhaled air particles and responding quickly to pathogens through the recruitment and activation of specific innate and adaptive immune cells, which play an essential role in the clearance of the virus and the development of antiviral immune memory cells [7].

In addition, in response to infection or inflammation, lung tissue forms an ectopic lymphoid tissue structure known as inducible bronchus-associated lymphoid tissue (iBALT) [14,15], which is quite similar to the germinal center (GC) structure in the lymph nodes, where B and T cells accumulate in clusters, and the outcome of this reaction can lead to an effective immune response and long-lasting memory cells. The cytokines and chemokines that induce and contribute to the formation of iBALT in COVID-19 are worth investigating. B-cell activation factor (BAFF) and A proliferation-inducing ligand (APRIL) are cytokines belonging to the tumor necrosis factor (TNF) superfamily, and both play an essential role in B-cell activation, differentiation, and antibody production [16]. The local expression of homeostatic chemokines, including C-X-C motif chemokine ligand 13 (CXCL13), chemokine (C–C motif) ligand 19 (CCL19), and CCL21, induces the formation of iBALT following influenza infection and plays an important role in providing an effective immune response [17].

Despite ongoing investigations on the development of systemic vaccines against SARS-CoV-2, there is no effective approved vaccine administered through the mucosal route. Thus, understanding the mechanism of how the local mucosal immune response against SARS-CoV-2 infection is mediated and enhances the formation of iBALT in the airways may help us to develop effective mucosal vaccines. In this review, we will discuss the role of the BAFF/APRIL system and hemostatic chemokines, including CXCL13, CCL19, and CCL21, considering the latest literature in this field of research.

## 2. Role of BAFF/APRIL System in B-Cell Response and Antibody Production

BAFF and APRIL are relatively newly discovered cytokines belonging to the TNF superfamily, and these cytokines share three receptors: the B-cell activation factor receptor (BAFF-R), the transmembrane activator and calcium-modulating cyclophilin ligand interactor (TACI), and the B-cell maturation antigen (BCMA) [18]. BAFF and APRIL interact with their cognate receptors at different affinities; BAFF has a high affinity for BAFF-r and TACI receptors and a low affinity for BCMA, whereas APRIL has a high affinity for BCMA and a moderate affinity for TACI but not BAFF-r [19]. The interaction of these cytokines with their cognate receptors can lead to a variety of immunological functions during B cell development, homeostasis, maturation, survival, differentiation, and antibody production [16]. BAFF and APRIL can be activated as membrane-bound proteins on the cell and tissue surfaces, or they can be processed as soluble molecules after cleavage from the cell surface via the furin convertase cleavage enzyme [20]. BAFF and APRIL are expressed by several cells and tissues, including neutrophils, macrophages, natural killer cells, monocytes, dendritic cells, B cells, eosinophils, basophils, fibroblasts, stromal cells, activated T cells, airway epithelial cells, autoimmune-disease-related cells, malignant blood cells, and solid tumors [16,21,22]. BAFF and APRIL receptors are predominantly expressed on the B cell surface with different expression patterns during B cell development. BAFF-R is expressed at high levels at the early stages of immature B cell subset development, while TACI is expressed by marginal zone B cells (MZ), memory B cells, and plasma cells, and BCMA is expressed by plasmablast plasma cells [23,24]. Both BAFF and APRIL play important roles in B cell survival and antibody production.

A study has shown that BAFF- and BAFF-r-deficient mice have very low peripheral blood B cell counts compared with control animals [25]. In addition, it has been reported that targeting BAFF with a monoclonal antibody (belimumab) in patients with systemic lupus erythematosus (SLE) results in the inhibition of B cell activation, proliferation, and differentiation, which in turn reduces the role of these cells in disease activity [26]. In addition, BAFF overexpression results in the enhanced progression of interstitial lung disease in common variable immunodeficiency (CVID), and the inhibition of BAFF results in the maintenance of disease progression [27]. Collectively, these findings suggest the importance of BAFF in the development and regulation of B cells. Additionally, BAFF can enhance the survival of B cells by regulating pro- and anti-apoptotic genes [16].

The administration of APRIL in mice resulted in increased B cell numbers [28]. Nevertheless, APRIL-deficient mice had normal B cells counts [29], suggesting that APRIL may be important for plasma B cells to survive after binding with BCMA at the late stages of their development [30]. Recently, we found that BAFF and APRIL expressions were increased significantly in patients with asthma compared to healthy controls, and the increased expression of BAFF-r/BCMA, but not TACI receptors, was associated with the increased expression of BAFF and APRIL, which may suggest a possible increased B-cell response to BAFF and APRIL [31].

Class switch recombination (CSR) is a biological mechanism in which B cells can be induced via two signals, namely, T-cell cytokines and the binding of CD40 and CD40 ligands [32]. Furthermore, BAFF and APRIL can induce CSR to produce a new class of antibodies through the heavy chain switch of the IgM to IgA, IgG, and IgE, directly without the help of T cells [33,34,35]. Moreover, both BAFF and APRIL were unable to stimulate CSR for IgA, but not IgG, synthesis in TACI-deficient mice [36,37]. Furthermore, the analysis of B cells from individuals with TACI mutations showed that human B cells were abolished in response to APRIL and BAFF during CSR induction to generate IgA but not IgG APRIL, and the interaction of BAFF with the BAFF-r receptor resulted in the production of both IgA and IgG [38]. In addition, the interaction of BAFF and APRIL with TACI in patients with chronic obstructive pulmonary disease prompted the CSR of IgA at the epithelial level [39]. BAFF and APRIL are expressed in the central nervous system following cytomegalovirus (CMV) and coronavirus infection and contribute to host resistance by sustaining virus-specific antibodies via the induction of TACI and BCMA receptors [40]. To date, no study has examined the functional role of the BAFF and APRIL systems in antibody production following SARS-CoV-2 infection; thus, further studies are warranted in this area of research.

## 3. Expression of BAFF/APRIL following Respiratory Viral Infection

BAFF and APRIL are expressed in response to respiratory viral infection. BAFF and APRIL expressions were elevated in mouse lungs after the influenza virus challenge [41,42]; however, it is not clear whether this increase can help to control influenza infection as neither APRIL-overexpression nor APRIL-deficient mice affected the host response to influenza virus in infected mice [42]. In contrast, a study on TACI-deficient mice after infection with the influenza virus failed to provide and sustain antibody titers in the mouse lungs and serum, resulting in short-lived plasma cells; thus, this may increase the susceptibility to re-infection with influenza virus [43].

Furthermore, BAFF and APRIL expressions were elevated in isolated infant lung epithelial cells following lethal respiratory syncytial virus (RSV) infection, suggesting that these cytokines are implicated in RSV infection. Furthermore, there was a positive association between the expression of BAFF and APRIL and the levels of antiviral IgA and IgM antibodies in nasopharyngeal secretions of RSV-infected infants [44]. Moreover, the expression of BAFF and APRIL after RSV infection depends on the induction of interferon β (IFN-β) through TLR3 activation [45]. Similarly, we also found that BAFF expression was elevated significantly after severe RSV infection in infants, as well as in cultured airway epithelial cells, and it further increased in the bronchoalveolar lavage fluid of infants with other viral respiratory infections, such as rhinovirus, metapneumovirus, influenza virus (H1N1), and bocavirus [46]. Although BAFF expression increased significantly after RSV infection, we did not detect increased APRIL expression after RSV infection in humans and mice [45,47]. However, APRIL expression was significantly increased after RSV infection, which plays an essential role in increased IgA production [44]. Different viruses can infect various cell types and tissues, and the expressions of BAFF and APRIL may differ from one cell type to another. Increased BAFF expression following viral infection is a common feature, but the type of virus and mechanism of BAFF expression differ depending on the cell type [48]. A recent study reported that BAFF expression was increased in the plasma of patients with COVID-19 and was positively correlated with B cell counts, whereas APRIL expression was increased in recovered patients, and the expression pattern of BAFF and APRIL was compatible with the IFN type I [49]. In addition, BAFF, but not APRIL, levels were increased more significantly in the sera of patients with severe COVID-19 than those in the sera of the non-severe group, suggesting robust B cell activation and corresponding high antibody response in patients with severe COVID-19 [50]. Although patients with COVID-19 have high plasma levels of BAFF, which is known to activate the generation and survival of plasmablasts, antibody titers are reduced over time [51]. Moreover, the immunohistochemistry analysis of patients with COVID-19 showed that BAFF was expressed in the airways compared to the healthy controls [52]. Furthermore, B cells from the bronchoalveolar lavage fluid (BAL) of patients with severe COVID-19 were mostly affected by BAFF rather than by APRIL [53]. A meta-analysis showed that increased BAFF expression may worsen the symptoms of COVID-19 [54]. However, the genetic analysis of patients with COVID-19 showed that BAFF-r was highly expressed in B cells, which reduced the risk of disease severity [36]. However, the precise role of the BAFF/APRIL system in SARS-CoV-2 infection remains unclear. Further studies are required to define the influence of these cytokines on the local B cell response in the lung and antibody production following SARS-CoV-2 infection.

## 4. Role of Pulmonary Homeostatic Chemokines CXCL13, CCL19, and CCL21 following Respiratory Viral Infection

The dysregulation of inflammatory cytokines and chemokines has been linked to COVID-19 severity [55]. Homeostatic chemokines, including CXCL13, CCL19, and CCL21, are expressed normally in secondary lymphoid organs constantly in a homeostatic balance [56]. The interaction of CXCL13 with its receptor CXCR5 can facilitate B cell homing and formation, as well as the maintenance of B cell follicles in both secondary lymphoid organs and iBALT [37,57]. In addition, CCR7 plays an essential role in the activation and recruitment of T cells from the blood to the T cell zone through high endothelial venules (HEVs) by binding to its ligands CCL19 and CCL21 [57].

Although these molecules are mainly expressed in secondary lymphoid organs, a previous study showed that these chemokines can be expressed in the lung after influenza infection and were shown to initiate and develop iBALT, where local B and T cells can respond to influenza and provide an effective host immune response [17]. However, we found that the RSV infection of mouse lungs resulted in significantly increased CXCL13 serum levels, but not CCL19 and CCL21 levels, compared to control mice. Tissue lung sections demonstrated that CXCL13 was localized in areas possibly establishing lymphoid aggregates, indicating the essential role of the local expression of these chemokines in response to RSV infection [47]. To date, no study has examined the presence of iBALT following SARS-CoV-2 infection.

Recent studies revealed that CXCL13 serum levels were elevated significantly in patients with severe COVID-19 who were admitted to the intensive care unit (ICU) compared to those with moderate disease, suggesting that measuring CXCL13 in the sera of patients with COVID-19 can be used as a predictive immunological marker for the severity of COVID-19 [58,59]. In addition, higher CXCL13 sera levels were detected in patients who survived COVID-19 compared to those in patients that did not survive, suggesting that CXL13 sera levels can be used as a novel predictor of the lethality of SARS-CoV-2 infection. Further, CXCL13 expression is associated with the increased production of antibodies against receptor binding domain (RBD) and S1 of SARS-CoV-2 [60]. Additionally, increased serum levels of CXCL13 were observed in patients with severe COVID-19 compared to those with moderate disease and healthy controls, and the increased expression of CXCL13 was associated with increased antibody-secreting cell (ASC) production and activated circulating T follicular helper cells (cTfh), suggesting that CXCL13 plays an essential role in increasing the GC activity of patients with COVID-19 [61]. Moreover, it has been found that patients with severe COVID-19 produce higher serum levels of CXCL13 and increased frequencies of ASCs and TFH cells, and increased production of the specific antibodies of the RBD of SARS-CoV-2, compared to those with moderate disease [62]. Furthermore, a study examining the serum levels of CXCL13 in convalescent COVID-19 at various severities showed that CXCL13 serum levels were elevated significantly in patients with severe COVID-19 compared to those in patients with mild, moderate, or asymptomatic disease. The increased CXCL13 levels were associated with the production of anti-S1 and -S2 proteins. Moreover, patients with severe COVID-19, but not those with asymptomatic or moderate disease, developed virus-specific GC B-cell responses associated with cTfh responses [63]. In addition, CXCL13 levels were increased significantly in the serum and BAL of rhesus macaques following SARS-CoV-2 infection [64]; however, the expression of CXCL13 in lung tissues and sections in human subjects has not yet been examined. Further studies are required to determine the role of CXCL13 in the initiation of local B cell responses during SARS-CoV-2 infection.

Furthermore, a previous in vivo study showed that CCL19 expression was higher in ferrets after challenge with SARS-CoV infection compared to that in controls [65]. In addition, CCL19 plasma levels were significantly elevated in patients with COVID-19 and were associated with poor symptoms and acute respiratory distress syndrome (ARDS), suggesting that CCL19 plasma levels can be used as an early prediction marker of worsening symptoms of COVD-19 [66]. In addition, CCL19 serum levels were increased remarkably in the lungs of mice following SARS-CoV-2 infection relative to control animals, implicating severe lung inflammation and diminished normal lung function [67]. Moreover, CCL19 expression was significantly increased among other cytokines in the lung tissue sections of patients with COVID-19, suggesting that CLL19 may be involved in the pathology of COVID-19 [68]. Additionally, CCL19 serum levels were increased in patients with mild, moderate, and critical COVID-19 relative to healthy controls, and the treatment of patients with COVID-19 with baricitinib resulted in decreased CLL19 expression, suggesting that CCL19 may contribute to the severity of COVID-19 [69,70]. Increased CCL19 plasma levels were detected in patients with COVID-19 treated in the ICU, which correlated with a high mortality rate [71]. However, a proteomic analysis of spleen tissue and BAL collected from patients with fatal COVID-19 showed that CCL19 expression was decreased, which was associated with decreased CD8+ T cell proportions [72]. Additionally, a study that examined gene expression and serum levels revealed that CCL19 was upregulated in patients with COVID-19 relative to healthy controls, and targeting CCL19 with bamlanivimab did not make a difference compared to the placebo group [73]. CCL19 serum concentrations did not differ between patients with COVID-19 who were treated in the ICU and those who remained stable over time [74]. These observations suggest that further studies are required to examine the role of the local expression of CCL19 in the recruitment of T cells during SARS-CoV-2 infection.

An in vitro study showed that HeLa cells challenged with the ORFa protein of SARS-CoV-2 produced several inflammatory chemokines, such as CCL19, CXCL13, and CCL21 [75]. In addition, the serum levels of CCL21 were found to be high in patients with COVID-19 who did not survive 12 days after SARS-CoV-2 infection [76]. Furthermore, human lung tissue sections from samples of patients with acute COVID-19 showed that CCL21 expression was weakly detectable but was upregulated in later phases of the disease, and the local expression of CCL21 recruited CCR7^+^ T cells and T follicular helper-like cells and can contribute to the formation of tertiary lymphoid structures in the lungs [77]. The precise role of CCL21 in the activation and impairment of proper T cells during SARS-CoV-2 infection remains unclear; thus, a subsequent study may define the precise role of this chemokine post-SASR-CoV-2 infection.

## 5. The Role of Mucosal Immunity during SARS-CoV-2 Infection

**Figure 1:**
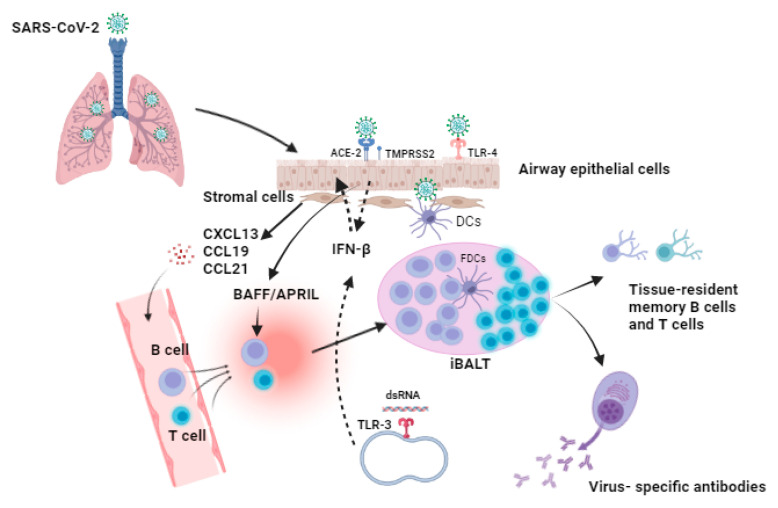
Proposed model for the formation of inducible bronchus-associated lymphoid tissue (iBALT) after SARS-CoV-2 infection. SARS-CoV-2 infection is initiated when the SARS-CoV-2 spike protein interacts with the angiotensin-converting enzyme two (ACE-2) receptor that is expressed on the surface of airway epithelial cells, and another essential host cell factor is the transmembrane protease serine 2 (TMPRSS2), which enhances the interaction of the spike protein with the receptor and facilitates the entry of the virus into the host cells. Additionally, toll-like receptor-4 (TLR-4) can recognize the spike protein of the virus, and endoplasmic TLR-3 can identify the viral double-stranded RNA (dsRNA) during replication. The host interaction with the virus may lead to the activation of type-1 interferons (IFNs) such as IFN-β, which can act as an antiviral agents to inhibit the viral replication and induce an antiviral state in the neighboring cells. Moreover, infected host cells produce a wide range of inflammatory cytokines and chemokines that can activate and recruit many immune cell types at the site of infection. They can activate dendritic cells (DCs) as well as stromal cells. Furthermore, during SARS-CoV-2 infection, local inflammation is initiated in the lung. Resting stromal cells, including fibroblasts, lymphatic cells, and vascular endothelial cells, are considered major sources that can produce homeostatic chemokines, such as CXCL13, CCL19, and CCL21, which further mediate the formation and maintenance of iBALT in the lungs via the facilitating and recruitment of B and T cells to the site of infection, where they can accumulate and arrange in a follicle germinal-like structure. In addition, follicular dendritic cells (FDCs) are located in the B-cell follicle, present specific antigens to the B cells, and enhance co-stimulatory signaling. Thus, FDCs are important in the maintenance of iBALT during infection. Furthermore, the cytokines BAFF and APRIL can be produced by airway epithelial cells upon viral infection and may support local B cell response, activation, differentiation, and antibody class switching that results in the production of specific antibodies against SARS-CoV-2 in the lungs, as well as the generation of memory B and T cells that can last for a long period of time.

Mucosal immunity is the most significant part of the immune system, playing an essential role in protecting the mucosae, which are in direct contact with inhaled pathogens. Thus, as part of mucosal immunity, there must be a careful balance between reducing the inflammation of tissues and ensuring that the body is capable of responding adequately to threats such as SARS-CoV-2 [78]. SARS-CoV-2 infection occurs in the upper respiratory tract (URT). The induction sites of the mucosal immune response in the nasopharynx-associated lymphoid tissue include the nasal epithelium, tonsils, and adenoids [79,80]. Furthermore, after crossing the mucosal barrier, viral particles bind to host cells through the interaction of the viral spike protein with the receptor ACE-2, facilitated by the co-receptor transmembrane protease serine 2 (TMPRSS2). Following this interaction, the virus fuses with the cellular membrane and releases its genomic RNA into the cytoplasm of the cells during viral replication [81,82] (Figure 1). Furthermore, endosomal TLR-3 receptors can identify dsRNA during SARS-CoV-2 replication, and this induces the activation of the transcription factors and the associated signaling pathways, including interferon-regulatory factors and nuclear factor-kappa B (NF-κB), which results in the activation of type I IFNs, as well as proinflammatory cytokines, and the expression of type I IFN can induce an antiviral state in uninfected cells [83]. Additionally, the spike protein of the virus can be recognized via toll-like receptor-4 (TLR-4) on the host cell surface, whose activation may enhance the cell surface expression of ACE2 and induce the expression of type I IFN and proinflammatory cytokines [84]. Thus, initial viral replication and shedding occur primarily in the URT, and viral replication precedes in the lower respiratory tract, where airway epithelial cells become infected and the patient may develop viremia [85].

Many studies have focused on profiling the immune response of patients with peripheral COVID-19. However, few studies have investigated the local immune response during SARS-CoV-2 infection. A transcriptome analysis of RNA nasopharyngeal samples collected from patients with COVID-19 revealed genomes related to cytokine-cytokine and chemokine interactions, as well as complement and coagulation cascades, and these gene sets are highly expressed in patients with severe COVID-19 [86]. Single-cell RAN analysis of BAL in COVID-19 showed that interactions between epithelial cells and cellular immune responses were associated with the severity of COVID-19 [87]. In addition, the single-cell RNA analysis of BAL of patients with COVID-19 revealed that CD8+ T-cell numbers were low in patients with severe COVID-19 compared to those with mild COVID-19, suggesting that immune responses were dysregulated and mild patients were more effectively controlled by viral replication than severe cases [88,89].

B-cell activation and antibody production play essential roles in the antiviral response. Specific IgM to SARS-CoV-2 antibody conversion was detected at approximately day 7 post-infection, and after the class switch, specific IgA and IgG were detected [89]. The investigation of convalescent plasma of patients with COVID-19 showed that IgG levels were expressed at a high level and were a more robust response than IgA, which persisted for a short time [90,91]. In addition, the durability of specific IgG antibodies in serum varies from 1 to 6 months and is correlated with disease severity [90,91,92,93]. However, it is still not fully understood how antibodies respond and persist in the mucosa following SARS-CoV-2 infection. Recent studies have found that mucosal levels of IgM and IgG are correlated in the serum [90,94]. However, another study showed a weak association between mucosal and serum IgA levels [90], which may suggest a different regulatory mechanism of antibody production in the mucosa.

## 6. Role of iBALT in Providing Protection against Viral Respiratory Infection

As the mucosal surface of the respiratory system is frequently exposed to environmental factors and stimuli, such as pathogens and allergens, these agents may induce the local inflammation of the lung, and aggregated B and T cells accumulate in follicles near the bronchi to form iBALT [95]. Additionally, iBALT formation can be initiated by the production of chemokines from stromal cells, which contribute to the recruitment and organization of lymphocyte iBALT [95]. Furthermore, iBALT is a transient structure that resembles and can act as a conventional GC, which is normally expressed in secondary lymphatic tissues [96]. Numerous viral respiratory infections, such as RSV, influenza, SARS coronavirus, and adenovirus infections, correlate with the formation of iBALT in the lungs [97,98,99,100]. iBALT formation occurred in mouse lungs following infection by the influenza virus, and the presence of iBALT supported the local immune response and enhanced production of neutralizing antibodies against influenza, while the disruption of iBLAT after two weeks of infection resulted in the decreased production of local IgA [101,102]. Furthermore, iBALT formation after influenza infection triggers the production of specific memory B cells in the lungs that are widely active against various strains of the influenza virus [103]. Additionally, when mice had pre-existing iBLAT, the production of neutralizing antibodies against influenza infection in the lung was faster and enhanced viral clearance compared to control animals [102,104]. Similarly, the presence of iBALT following SARS coronavirus infection in mice improved the rate of viral clearance and increased the production of virus-specific antibodies [104]. Moreover, mice infected with pneumovirus developed iBLAT and produced more efficient virus-specific antibodies with enhanced viral clearance in the lungs, which decreased the morbidity post infection [105].

## 7. Mucosal Vaccination against SARS-CoV-2 Infection

The current systematic vaccines for COVID-19 are delivered by injection and designed to induce an IgG response that can prevent viremia and COVID-19 symptoms [4]. Conversely, the ability of systemic respiratory vaccines to provide full protection upon respiratory viral infection is limited, suggesting the need for a protective mucosal vaccine as SARS-CoV-2 infection starts first from the mucosal respiratory system. Thus, to prevent viral replication in primary mucosal cells, the sufficient production of local secretory IgA (SIgA) is required, which can be induced via a vaccine administered through the mucosal route [4]. A previous study showed that an intranasal vaccine spray (FluMist) against influenza can induce higher levels of local SIgA in airways relative to injectable vaccines [106]. Furthermore, the intranasal immunization of mice with chimpanzee Ad-vectored vaccine encoding SARS-CoV-2 proteins (spike-1, nucleocapsid, and RdRp) offered a strong local and systemic antibody response and activated an innate response and mucosal tissue-resident memory T cells compared to intramuscular immunization, suggesting that the mucosal immunization route can provide extra protection against SARS-CoV-2 and its variants of concern (VOC), including VOC; B.1.1.7; and B.1.351 [107].

An early study showed that the pulmonary administration of nanoparticles in protein cages results in the formation of iBALT in the airways, which plays an essential role in promoting the production of neutralizing virus-specific antibodies, improving viral clearance, and reducing morbidity as well as lung pathology [104]. The intranasal administration of Ag85B-expressing human parainfluenza type 2 virus (Ag85B-hPIV2) in mice showed that iBALT was formed in the lungs of mice, which played an essential role in the induction and activation of a specific immune response against tuberculosis (TB) compared to that in control mice. Moreover, upon the disruption of iBALT, immune responses (such as production of IFN-γ) and IgA levels were reduced in the mice lungs [108], suggesting the importance of iBALT in protecting lungs, along with the anti-tuberculosis mucosal vaccine.

Furthermore, it has been reported that the aerosol immunization of macaques with attenuated *Mycobacterium tuberculosis* leads to the formation of iBALT, which induces specific immune responses and central robust CD4+ and CD8+ T cell memory responses in the lung and offers protection for infected macaques against lethal TB challenge [109]. It has been speculated that the effective killing of influenza-infected epithelial cells is enhanced by the formation of iBALT [110]. Moreover, a study in a murine model of Tularemia showed that an intranasal vaccine with a neisserial recombinant (PorB) adjuvant candidate induced the formation of iBALT, which may play an essential role in the regulation of the local immune response and provide long-term protection [111]. A recent study of a modified vaccinia Ankara vector-based vaccine (MAV/s) encoding for the spike protein of SARS-CoV-2 was administered intranasally to macaques, which resulted in the robust production of neutralizing antibodies and generated an effective CD8+ T cell response in macaques following SARS-CoV-2 infection, protecting animals from viral replication in the lungs as early as two days after SARS-CoV-2 challenge compared to control animals [112]. Additionally, the immunostaining of vaccinated mouse lung tissues showed the formation of iBALT compared to naïve mice, suggesting a local effective B and T cell immune response post MAV/s vaccination [112], which may contribute to a quick immune response in the airways following SARS-CoV-2 infection.

Furthermore, BAFF can be induced following viral respiratory infection of the airways and has been suggested to induce local B cell responses and specific antibody production [44,47,48]. Chickens vaccinated with an inactivated infectious bursal disease virus vaccine (IBDV) in combination with immunostimulant soluble BAFF were protected from developing the infectious bursal disease compared to unvaccinated chickens [113]. Similarly, compared with control animals, mice vaccinated with inactive membrane BAFF-rabies virus showed virus-specific antibody titers that were higher and increased at a faster rate [114]. Furthermore, a recent study used virus-like particle technology to generate a multi-vaccine for seasonal influenza infection (H5H7 and H1H5H7) via the fusion of BAFF and APRIL and the incorporation of the influenza hemagglutinin (HA)-encoding gene [115]. Taken together, these findings suggest that BAFF/APRIL can be used as a vaccine adjuvant to enhance mucosal vaccination against SARS-CoV-2 infection.

## 8. Conclusions

Mucosal surfaces are the main routes of entry for SARS-CoV-2; thus, understanding the early events of how various immune cells respond and are recruited during SARS-CoV-2 infection, as well as how the host interaction in the lungs may differ among patients with COVID-19 with different disease severities, is important. Mucosal vaccines (nasal, spray, and oral) may have good outcomes and provide effective responses against SARS-CoV-2 infection as they can induce local and systemic immune responses. Furthermore, the delivery route of mucosal vaccines is rapid and can activate several immune subsystems. The presence of iBALT post-SARS-CoV infection and the local production of BAFF and APRIL during infection may play an essential role in regulating and activating a local immune response, increasing the production of specific antibodies, including IgG and IgA in the airways, and inducing long-lasting resident B and T memory cells that can offer subsequent protection. Although the current review highlights the importance of iBALT in enhancing the efficiency of the local mucosal immune response to viral respiratory infection, other studies have found that iBALT formation is implicated in the development of autoimmune diseases; thus, further studies are required to define and understand the biological mechanisms that control and regulate iBALT formation and function. Collectively, we suggest that BAFF/APRIL or CXCL13, combined with the spike protein-encoding mRNA of SARS-CoV-2, can be used as an immunostimulant adjuvant as an mRNA mucosal vaccine to enhance the local mucosal immune response against SARS-CoV-2 infection.

## Data Availability

Not applicable.
